# Partial purification and characterization of a thermostable xylanase from *Bacillus licheniformis* isolated from hot water geyser

**DOI:** 10.1186/s43141-022-00333-4

**Published:** 2022-03-29

**Authors:** Girisha Malhotra, Shilpa S. Chapadgaonkar

**Affiliations:** 1 Manav Rachna International Institute of Research and Studies, Faridabad, Haryana India; 2 MIT World Peace University, Kothrud, Pune, Maharashtra India

**Keywords:** Xylanase, Thermostable, *Bacillus*, Enzyme purification, Enzyme characterization, Gel filtration chromatography

## Abstract

**Background:**

Thermo-alkali stable xylanases were purified from the extracellular broth of newly isolated *Bacillus licheniformis* strain produced in a 5-L stirred-tank bioreactor with wheat bran as a carbon source.

**Results:**

A high degree of purity was achieved using size exclusion chromatography resulting in 16-fold purification and 69% recovery for fraction 5 which had the highest activity. The recovery obtained after pooling fractions 5 and 6 was 99%. The *K*_*m*_ value of xylanase was calculated as 0.05 mM, and *V*_max_ was 125 μmol/min/mg protein.

**Conclusion:**

Purified xylanase had a high thermal and pH stability. Xylanase was found to be suitable for application in the de-inking of paper and for saccharification of lignocellulosic waste biomass.

## Background

Xylanase is one of the most important industrial enzymes. It is extensively used in poultry and animal feed, paper and pulp, fruit juice, and fine chemical industries [1]. Xylanases for industrial applications can be isolated from several species of bacteria and fungi. Thermal and pH stability are the two most important properties of xylanases used in industry. In our previously reported work, we have reported the isolation and characterization of thermo-alkalophilic xylanase from *Bacillus licheniformis* [2]. In the present paper, the partial purification and characterization of xylanase from *Bacillus licheniformis* has been described.

**Fig. 1:**
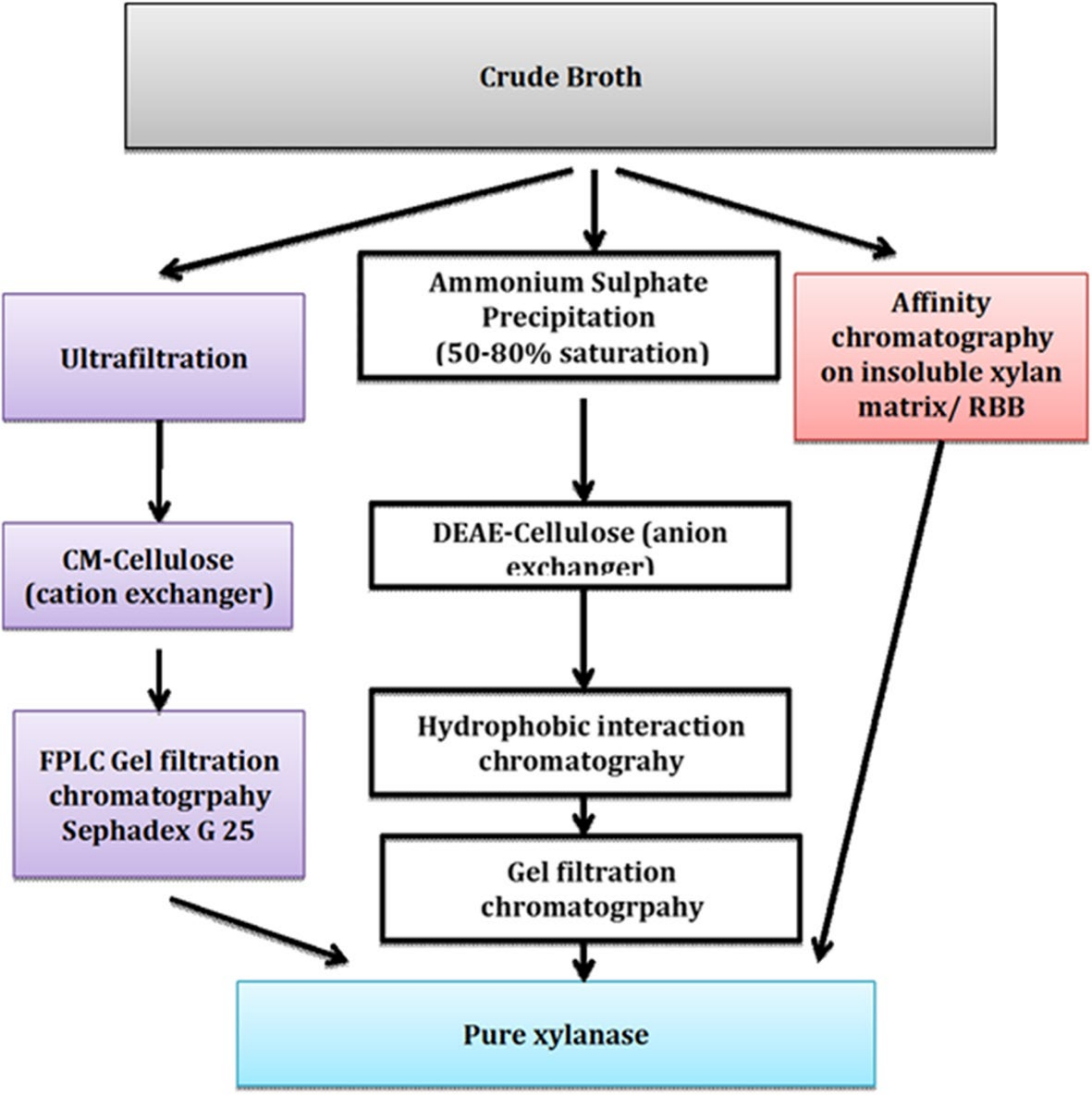
Xylanase purification strategies employed by various researchers

Xylanases have been purified using various strategies including ultrafiltration [3], ion-exchange chromatography [4], gel filtration chromatography [5], and affinity chromatography [6]. The level of purity desired is dependent on the field of application. Usually, complete purification of xylanases requires multiple steps consisting of two or more of the above techniques. Popular approaches for the purification of xylanases have been summarized in Fig. 1. Multi-step purification strategies are costly due to the product loss and costs incurred per step which in turn proves to be a deterrent for its application. In our previous work, we have attempted purification of xylanase using an aqueous two-phase system and product concentration using ammonium sulfate precipitation [7]. In order to improve the economy of xylanase application, we report here the purification of xylanase in a single-step strategy. The isolated *Bacillus licheniformis* strain was cultivated in a 5-L stirred-tank reactor using an optimized production medium, and operating conditions were optimized as described elsewhere [8].

## Methods

### Microbial strains and xylanase production

The newly isolated *Bacillus licheniformis* strain was routinely maintained in a nutrient agar medium at pH 7.0 and temperature 35 °C. The glycerol stock cultures and working stock cultures were revived regularly. The production medium had been optimized to maximize the yield of xylanase (Malhotra and Chapadgaonkar 2020). The optimized conditions used for the production process were as follows: (i) pH 6, (ii) culture temperature 35 °C, (iii) concentration of xylan 2% w/v, and (iv) concentration of wheat bran 2.5% w/v. The broth was centrifuged and the supernatant was used for further purification of xylanase.

### Production of xylanase in a 5-L stirred-tank reactor

Bioage 5-L stirred-tank bioreactor was used for xylanase production with previously optimized process conditions. The optimized xylanase production medium was used as described before. The reactor was sterilized by autoclaving for 1 h. After sterilization, the reactor vessel was connected to the control unit and recirculated the cooling water bath and allowed to cool to the process temperature. It was inoculated with 10% v/v of actively growing culture of the strain of *Bacillus licheniformis*. The reactor was operated at the optimized process parameters viz. pH 6.0, temperature 35 °C, RPM 125, and dissolved oxygen concentration (DO) of 60%. The reactor was harvested at peak xylanase activity achieved after 48h of the culture. The broth was centrifuged at 10,000 RPM for 10 min to remove the cells and stored appropriately for further studies.

### Assay of xylanase and protein concentration

Xylanase activity was determined by the 3,5-dinitrosalicylic acid (DNSA) method given by Miller 1959 [9]. In brief, the culture broth was first centrifuged at 10,000 rpm for 15 min at 10 °C to separate the cells. 1% w/v beechwood xylan (Himedia MB141-10G) prepared in 0.05M sodium citrate buffer was used as substrate. One milliliter of the substrate was incubated with 500μl of the enzyme (supernatant obtained from centrifuged culture broth) at 50 °C for 15 min. The reaction was terminated by the addition of 3 ml of DNSA, and the mixture was boiled for 10 min in a water bath. The absorbance was measured at 540 nm after cooling the mixture. Xylose was used as standard. One unit of xylanase activity (U) is defined as the amount of enzyme that liberates 1 μmol of reducing sugar-xylose per min under the standard assay conditions. Protein concentration was measured by lowry's method [10].

$$Xylanase\ activity\ U/ ml=\frac{\upmu moles\ of\ xylose\ liberated\times Dilution\ factor}{volume\ of\ enzyme\ used\times time\ of\ reaction}$$

### Lyophilization

The culture supernatant was concentrated by lyophilization in a lyophilizer (Allied Frost) for 6 h. The lyophilized sample was resuspended in phosphate-buffered saline (PBS 0.5M), pH 7.2, and then used for further studies.

### Gel filtration

Sephadex G-25 (0.2 × 5) column was used for size-exclusion or gel-permeation chromatography. The column was pre-equilibrated with PBS (pH 7.0), and lyophilized culture broth (200 μl) was loaded carefully on the column. The elution was performed using PBS as given before. Five hundred microliters of fractions were collected and subjected to spectrophotometric protein and xylanase determination.

### Molecular mass determination by SDS PAGE and zymogram analysis

The molecular weight of xylanase was estimated using 12% polyacrylamide gels by SDS PAGE [11]. The gel was visualized by Coomassie Brilliant Blue R-250 staining. Broad range protein molecular weight marker Bangalore GeNei, India, was used as standard.

Zymogram analysis was carried out using 0.1% beechwood xylan (w/v) incorporated into the polyacrylamide gel. The native PAGE was run without using SDS in the gel as well as in the running buffer. After the run, the gel was washed 4–5 times with distilled water for 30 min at 4°C and incubated for 24 h at room temperature in PBS. After incubation, the gel was immersed in 0.1% Congo red solution. The stained gel was then washed with an excess of 1M NaCl. The position of xylanase could be obtained as a clear band in the gel. After destaining the gel, it was being treated with 0.5% v/v acetic acid to increase the contrast between the bands and background.

### Thermo and pH optima

Temperature optima of the partially purified xylanase were determined by changing the incubation temperature while performing the enzyme assay (Fig. 4). Similarly, pH optima of the partially purified xylanase were obtained by incubating the partially purified enzyme in 0.05M buffer solutions in pH range 6.5–9.5 (citrate in the range of 4.5–6.5, phosphate in the range 7.5–8.5, and tris buffer in the 9.5 range) and determination of activity at that pH at 50 °C.

### Study of kinetics of xylanase activity

Initial reaction rates using beechwood xylan as a substrate were determined at a concentration of 0.5–10 mg/ml in 50 mM phosphate buffer (pH 7.0) at 50 °C. The assay method has been detailed in the previous section. The kinetic constants, *K*_*m*_ and *V*_max_ were estimated using the linear regression method of the Lineweaver-Burk plot.

## Results

The culture supernatant was concentrated by lyophilization and resuspended in PBS 0.05M, pH 7.2 in a lyophilizer (Allied Frost) for 6 h. The lyophilized sample was further purified by size exclusion chromatography. The comparison of protein content and xylanase activity before and after lyophillization has been given in Table 1.

**Table 1:** Concentration of xylanase in culture broth by lyophilization

Sample	Xylanase activity U/ml	Protein concentration mg/ml	Specific activity U/mg
Culture broth	157	0.56	280.36
Lyophilized supernatant	560	4.74	118.14

### Gel filtration

**Fig. 2:**
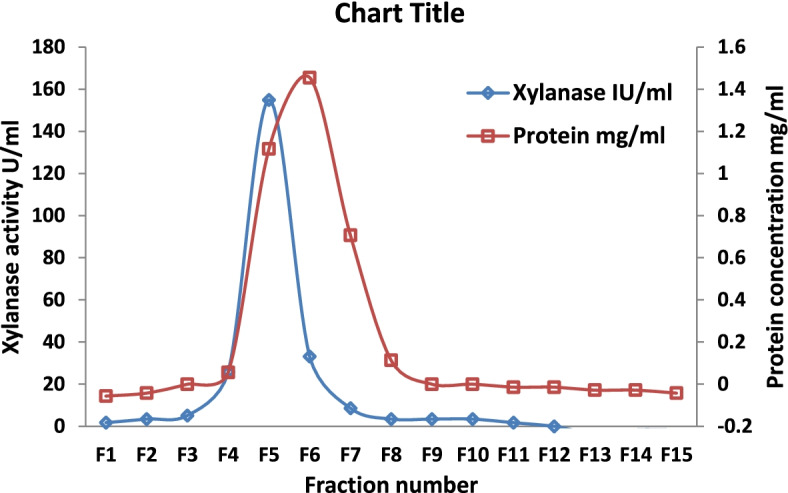
Xylanase protein peaks in gel chromatography

Gel filtration chromatography gave a high degree of xylanase purification in a single step (Fig. 2 and Table 2). The fraction numbers 5 and 6 exhibited high xylanase activities while very low activity was present in the other fractions. Gel filtration chromatography was successful in achieving 13.1-fold purification and a 69% (99% total including fractions 5 and 6) recovery of xylanase in a single step.

**Table 2:** Single-step purification of xylanase by Sephadex G-25 gel filtration chromatography

	Total xylanase activity (U)	Protein concentration (mg)	Specific activity (U/mg)	Fold purification	% Recovery
Lyophilized concentrate	112	0.95	117.89	1.0	100
Fraction 5	78	0.05	1560.00	13.2	69.2
Fraction 6	33	0.064	515.62	4.4	29.6

### Characterization of purified xylanase

**Fig. 3:**
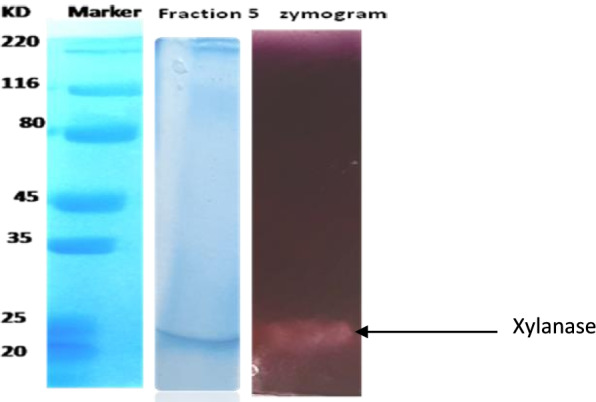
SDS-PAGE and zymogram analysis of purified xylanase

**Fig. 4:**
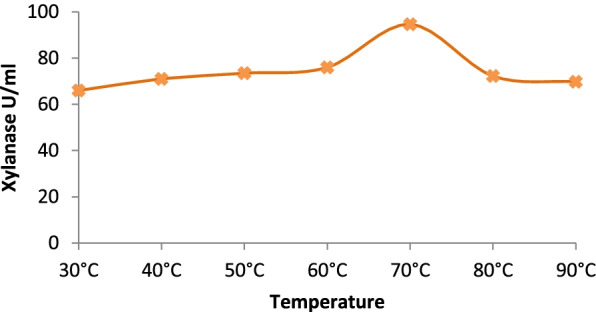
Temperature optima of purified xylanase

**Fig. 5:**
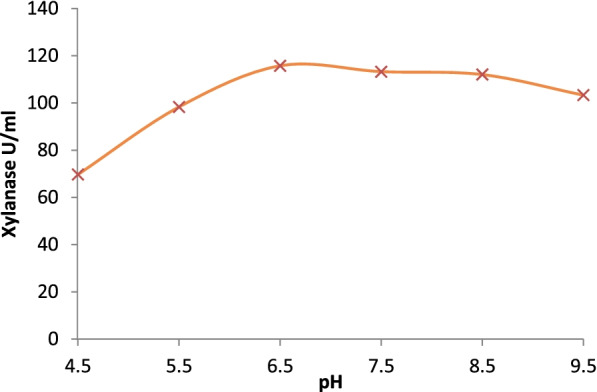
pH optima of purified xylanase

**Fig. 6:**
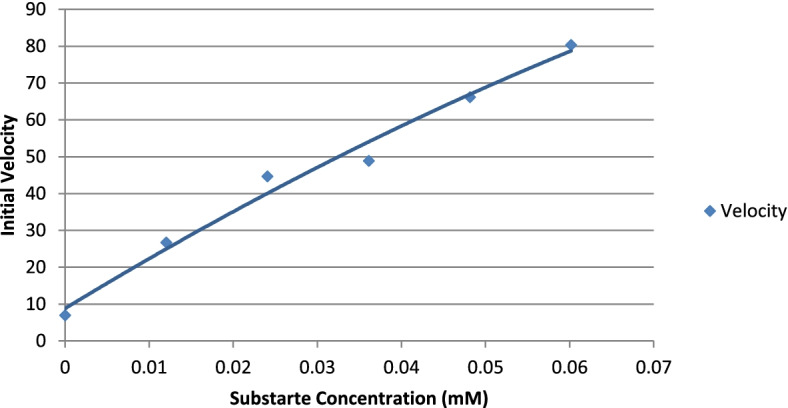
Initial reaction rate vs substrate concentration

**Fig. 7:**
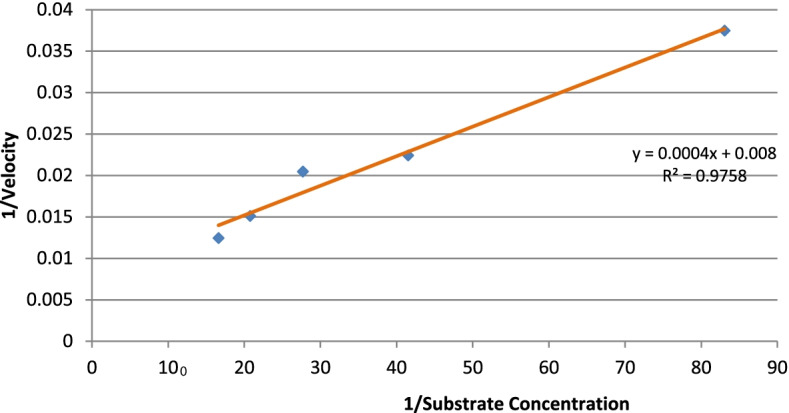
Lineweaver-Burk plot for the determination of *K*_*m*_ and *V*_max_

The molecular weight of the xylanase active band was confirmed by zymogram analysis in native PAGE (Fig. 3). Xylanase had a molecular weight of around 25 kDa. The purified xylanase was thermostable and showed optimum activity at 70 °C and remained highly active between a broad pH range of 6.5 and 9.5 (Figs. 4 and 5). When the initial rate of reaction was plotted against substrate concentration, it resulted in a typical hyperbolic profile (Fig. 6). The reaction rate increased with an increase in the concentration of xylan up to 10 mg/ml, beyond which there was a little change in the reaction rate (Fig. 6). Lineweaver-Burk plot of the enzymatic reaction was also plotted to determine *K*_*m*_ and *V*_max_. The *K*_*m*_ value of xylanase was calculated as 0.05 mM and *V*_max_ was 125 μmol/min/mg protein (Fig. 7).

## Discussion

The purification of enzymes is essential to ensure high activity, specificity, and stability. However, it is the most cost-intensive process due to the losses that occur with each step of the enzyme processing. Therefore, designing of purification process giving a high degree of purification in a smaller number of steps is highly desirable. In this paper, one such strategy has been described where xylanase purification could be achieved in a single step. Here, the size exclusion chromatography with Sephadex G25 was able to purify the xylanase 13.2-fold purification and 69% recovery. Previous work on a single-step purification of xylanases has involved either ion-exchange chromatography with CM Sephadex or DEAE Sephadex ion-exchange chromatography. Sanghi et al. [4] had purified xylanase by first fractionating the enzyme using ammonium sulfate purification and subsequently gel filtration chromatography using Sephadex G-100 column [12]. Verma and Satyanarayana xylanase possessed excellent thermal and alkali stability which is highly desirable for its applications especially in the paper and pulp industry [13]. It can be said that the single-step purification strategy owes its success to the low molecular weight of about 25 kDa.

## Conclusion

A high degree of purity was achieved using size exclusion chromatography resulting in 16-fold purification and 69% recovery and if we pool the fractions 5 and 6 and then almost 99% recovery can be obtained. Purified xylanase had high thermal and pH stability. Xylanase was found to be suitable for application in the de-inking of paper and for saccharification of lignocellulosic waste biomass. The *K*_*m*_ value of xylanase was calculated as 0.05 mM while *V*_max_ was 125 μmol/min/mg protein.

## Data Availability

The datasets used and/or analyzed during the current study are available from the corresponding author on reasonable request.

## Abbreviations

RPM: Revolutions per minute
DO: Dissolved oxygen
DNSA: Dinitrosalicylic acid
